# “Wrong, but Useful”: Negotiating Uncertainty in Infectious Disease Modelling

**DOI:** 10.1371/journal.pone.0076277

**Published:** 2013-10-16

**Authors:** Robert M. Christley, Maggie Mort, Brian Wynne, Jonathan M. Wastling, A. Louise Heathwaite, Roger Pickup, Zoë Austin, Sophia M. Latham

**Affiliations:** 1 Institute of Infection and Global Health, University of Liverpool, Neston, Cheshire, United Kingdom; 2 National Consortium for Zoonosis Research, Neston, Cheshire, United Kingdom; 3 Department of Sociology and School of Medicine, Lancaster University, Lancaster, United Kingdom; 4 Centre for Economic and Social Aspects of Genomics, Lancaster University, Lancaster, Lancaster, United Kingdom; 5 Lancaster Environment Centre, Lancaster University, Lancaster, United Kingdom; 6 Biomedical and Life Sciences Division, Lancaster University, Lancaster, United Kingdom; Université Catholique de Louvain, Belgium

## Abstract

For infectious disease dynamical models to inform policy for containment of infectious diseases the models must be able to predict; however, it is well recognised that such prediction will never be perfect. Nevertheless, the consensus is that although models are uncertain, some may yet inform effective action. This assumes that the *quality* of a model can be ascertained in order to evaluate sufficiently model uncertainties, and to decide whether or not, or in what ways or under what conditions, the model should be ‘used’. We examined uncertainty in modelling, utilising a range of data: interviews with scientists, policy-makers and advisors, and analysis of policy documents, scientific publications and reports of major inquiries into key livestock epidemics. We show that the discourse of uncertainty in infectious disease models is multi-layered, flexible, contingent, embedded in context and plays a critical role in negotiating model credibility. We argue that usability and stability of a model is an outcome of the negotiation that occurs within the networks and discourses surrounding it. This negotiation employs a range of discursive devices that renders uncertainty in infectious disease modelling a plastic quality that is amenable to ‘interpretive flexibility’. The utility of models in the face of uncertainty is a function of this flexibility, the negotiation this allows, and the contexts in which model outputs are framed and interpreted in the decision making process. We contend that rather than being based predominantly on beliefs about quality, the usefulness and authority of a model may at times be primarily based on its functional status within the broad social and political environment in which it acts.

## Introduction

Over the last few decades infectious disease dynamical modelling has attained a central role in contingency planning for outbreaks of human and livestock diseases and in guiding policy responses in the face of epidemics [1], [2]. Such modelling activities may be commissioned by governments, or may be developed independently by researchers. However, in each case a strong motivation is to guide some form of action to prevent or respond to a disease event or threat. Some recent examples include modelling to inform policy decisions for human and animal diseases such as SARS, H1N1 swine influenza, H5N1 Avian influenza, HIV, foot-and-mouth disease, classical swine fever and bluetongue.

A key feature of many of these epidemics is that they are emerging or re-emerging [3], [4] and thus have limited or no precedents within the time and place of interest. Such situations are simultaneously proposed as being ideally suited to the application of modelling and problematic for modelling. That is, whilst modelling may be proposed to provide clarity in complex and rapidly emerging situations [5], there is often no “off the shelf” model available, the system may be poorly defined and understood, input data may be limited, there is likely to be limited replicate data for validation and there may be unrealistic pressure for rapid results.

Key to the role of models in informing policy for containment of specific diseases is the requirement for the model to be able to predict [6] although it is well recognised that such prediction can never be perfect [7]. That is, models should not aim for ‘certain knowledge’ but rather should provide adequate ‘approximations’ of a real world. The lacunae that prevent the model approximations from attaining certainty include a wide range of factors that we refer to as *uncertainties*. A range of classifications of uncertainty has emerged in the natural and social sciences [8]. A key distinction can be made between *weak uncertainties* (also known as ‘probabilistic’ uncertainties, ‘statistical’ uncertainties or ‘risks’) that can be expressed in probabilistic terms, and *strong uncertainties* (also called ‘scenario’ uncertainties or just ‘uncertainty’) where a range of possible outcomes may be known (or may remain unknown in the case of *ignorance*), but where probabilities cannot be ascribed to these. An example of weak uncertainty could include the estimation of the probability of transmission of an infectious agent from an infectious individual to a susceptible individual given a particular form of contact between them. This probability is bounded by 0 and 1, but its precise value, or even the most likely range of values, remains uncertain. An example of strong uncertainty could include questions relating to the systems involved, for example: ‘how should the systems be conceptualised in a model?’ and ‘what, and whose, priorities should dictate the goals of interventions, and how are the effects of such interventions best assessed?’ A further key distinction can be made between reducible (or epistemic) uncertainty and irreducible (ontological) uncertainty [9]. Some of the former represent a failure of practice (or, at least, a limitation) which can be resolved through, for example, improved measurement or further research, but other elements may reflect what has been defined as the essential finitism of scientific knowledge [10]. The latter reflects a fundamental inability to know some things and results in outcomes that defy prediction because causal chains and networks are open or contingent [8].

In this paper, we agree with Wynne [11] that there “is no fixed level of uncertainty ‘out there’, but different interacting perceptions of how much, and of what shape and meaning it has”. Uncertainty is well recognised in modelling practice and its influence is expressed in the well-known mantra: “All models are wrong, but some are useful” [12]. For example, forms of this saying can be found in infectious disease modelling textbooks (for example [7]) and in statements by policy makers (quoted in [13]).

Whilst this notion of being *wrong but useful* recognises the uncertainty inherent in modelling, it also assumes the uncertainties in individual models can be sufficiently stabilised to permit use of the model predictions for valid decision-making. The consensus appears to be that although models are uncertain, some may yet inform action. An implicit qualification may be suggested here – that with such qualities, models may inform effective action, so long as they are interpreted and used appropriately. Such statements assume what sociologists of science policy describe as a reflexive capability on the part of modellers and their policy users – that the *quality* of a model be ascertained in order to decide whether or not, or in what ways or under what conditions, it should be ‘used’. However, model uncertainties are not calculable – there are no *units of uncertainty*. Only some particular uncertainties can be quantified, and even these may be uncertain.

We argue that the utility of a model is negotiated in practice within the networks and discourses surrounding it. So that while the common view is that “Simulation models are virtual worlds which aim to mimic the real world…” even if “…by necessity they are approximations of the real world” [14], a model can never be a faithful reflection of nature or of a human-nature system. A model is produced by individuals who must make a multitude of decisions during its creation and who bring a range of assumptions, social and natural, to defining the system of interest, and to defining the actors and their relationships, which compose it. Hence, a model is not simply the *in silico* representation of a system, free from human influence, but rather is socially produced within the discourses of its representation. Discourse here encompasses text and spoken word as well as algebraic, diagrammatic and other representations of models, including tables, figures and images depicting results. This discourse occurs in informal conversation, formal presentation, and in all communication with colleagues, policy makers and lay communities, without which the model would not exist in any meaningful way and could not be socially active as a tool.

The aim of this paper was to explore how models come to be stabilised in the face of multiple uncertainties. In doing so we show that modelling discourse is multi-layered, flexible, contingent, embedded in context and plays a critical role in negotiating credibility. Here, negotiation is a process that operates in three senses: the modeller tries to *navigate* a way through a set of articulated uncertainties; to *convey* the efficacy of the methods used to mitigate these uncertainties; and, in doing so, *agree* with others a co-produced understanding of the level of uncertainty present in the model. We contend that negotiation enables production of a more coherent model by both addressing and obscuring uncertainties. It is, we argue, through this that models are able to be presented as valid and coherent visions of ‘reality’ and hence gain authority to guide action. This is a necessary practice in order to invest models with sufficient authority that they can act within the decision making process; it is the means by which some models may be judged useful, and others not.

Our interdisciplinary analysis examines methods through which model uncertainty may be negotiated, beginning with what it is that models should do in terms of prediction. Subsequently we dissect the technical practices of model definition, design and testing respectively, in order to illustrate the flexibility with which issues of uncertainty are negotiated in infectious disease model production. Finally, we attend to the interaction between modelling and policy-making and explore the role of negotiation in this process.

## Methods

### Ethical statement

This study was approved by the Lancaster University Research Ethics Committee. Participants were provided with information describing the project several days prior to the interview and the purpose of the project was further discussed before each interview commenced. Participants were informed that their interview transcripts would be anonymised, kept securely and that any material potentially leading to identification would be removed. Consent forms, signed by all participants, specified that they could terminate the interviews, or withdraw from the study, at any time without providing a reason.

### Study design

The analysis presented in this paper draws on a 3-year interdisciplinary research programme addressing the social, technological and natural dynamics of animal disease management across a range of policy scales (http://www.relulostintranslation.co.uk/). The project team comprised veterinary scientists and epidemiologists, sociologists, microbiologists and environmental scientists. We utilised a range of primary and secondary data including 24 interviews with scientists (including mathematical modellers, infectious disease biologists, public health and veterinary scientists), policy-makers, advisors and other users of model results, as well as analysis of policy documentation, scientific publications, textbooks and reports of inquiries into key animal epidemics. Interviews were conducted in person, lasting between (approximately) 30 and 150 minutes. Interviews were semi-structured and included open-ended questions to elicit discussion around complex topics and areas of interest. Because this research investigated a range of issues related to disease containment and management, participants were necessarily drawn from a wide range of backgrounds. Hence, while some interviews focused on issues directly related to dynamical infectious disease modelling, others only briefly addressed this topic. Policy documents, scientific publications and reports of inquiries were identified using a combination of Internet search engines (Google and Google Scholar), online databases (Pubmed, Web of Knowledge and Scopus), citations in identified key publications and by recommendation from study participants and the project's multi-agency steering group.

### Analysis

All interviews were recorded and transcribed verbatim. The transcripts and other documents drawn on were subjected to multiple readings and analysed using an open coding method by the first author. This entailed line-by-line coding, examining the content, structure and the explicit and implicit meanings within the text. Basic analytical codes were developed from this fine level of data analysis, which involved both inductive (arising from the content of the text) and deductive (questioning the implicit points within the narrative) approaches. As the analysis continued further routes of enquiry emerged from the data itself and from theory derived in similar studies from within the field of Science and Technology Studies (STS, see below). Results were discussed in detail among the first three authors and at in-depth cross-disciplinary meetings with all authors.

Although not a specific intention at the outset, the insights in this paper predominantly relate to models of foot-and-mouth disease (FMD), particularly to those relevant to the UK 2001 FMD outbreak, in which modelling had a central and controversial role.

### Theory underpinning the analysis

In our analysis, we draw on insights from the field of STS and in particular on three key studies of scientific discourse: Gilbert and Mulkay [15], Pinch et al [16] and Singleton [17]. Gilbert and Mulkay [15] draw upon interviews and observations with biochemists, exposing distinctive forms of scientific accounting (the *empiricist* and the *contingent* repertoires) and their selective use in discursive exchanges between competing schools of thought. They found that formal scientific research literature is dominated by the empiricist repertoire in which data are given priority over the actions of the author. In contrast, the contingent repertoire is more informal and idiosyncratic, and insights are revealed as being dependent on speculative understandings, prior intellectual commitments, personal characteristics, tacit skills, social ties and group membership.

Pinch et al [16] analyse discourse surrounding the proposed, and controversial, introduction of clinical budgeting in the UK National Health Service in the mid 1980s. They identify two rhetorical patterns that, in part, parallel the empiricist and contingent repertoires of Gilbert and Mulkay [15] but which ascribe greater function to forms of discourse. The strong program (“hard sell”) draws on an empiricist repertoire, in which health economics is treated as a rational calculator demanding radical change. In contrast, the weak program (“soft sell”) draws on a more contingent repertoire, in which the controversial methodology is presented as user friendly, helpful to practitioners and not involving radical changes. Pinch et al [16] also explore discourse around the testing of new technology to reveal the role of these rhetorical patterns in production of successful test outcomes.

Singleton's study [17] of the discourse of laboratory workers in the UK national cervical screening program identifies both a “triumphant discourse about the successful introduction and expansion” of the screening program that prevents the progression of cervical cancer and saves lives and “reference to continued mortality and persistent failure…which constructs [screening] as problematic and ineffective”. Hence, screening is characterised by “instability” and “multiple identities”, yet rather than undermining the program, or the role of the laboratory, Singleton illustrates how this instability and multiplicity contributes to the continuity of the program and strengthens claims regarding the role of the laboratory in the program.

## Results and Discussion

### Models and prediction

In this paper we are particularly interested in those models that are used for prediction where issues of uncertainty are central to claims about the utility of model results. Many models of foot-and-mouth disease (FMD) in the UK have clear claims of a predictive role, and/or are interpreted to have such a role; for example such claims occur in in the reports of the major inquiries into the 2001 epidemic [18]–[20]; and in scientific papers describing models (e.g. [21]–[25]).

There is, however, evidence of confusion regarding the role of models in prediction, and in what is thought reasonable to expect of their predictive ability. At one level there is open recognition that models cannot accurately and explicitly reconstruct actual epidemic events:

**E1 (Report)**

Despite the advances in modelling that will arise in the coming decades, models will *never* be able to accurately predict if, or when, a particular person, farm or community will become infected. This is for two reasons: (i) the transmission of infection is a stochastic process, such that no two epidemics are identical; (ii) models will always be an approximation, and rare or unforeseen behavioural events can have a significant impact on the disease dynamics [italics in original] [26].

Here, two contingent effects are highlighted. The first recognises the indeterminacy of the system being modelled (at least at the level of the individual person, farm or group); the second highlights the uncertainty (perhaps ignorance) present in the system contextualisation, model composition and parameterisation and an inability to plan for surprise events. This mirrors the distinction noted earlier between ontological (real system) and epistemic (knowledge of it) uncertainty.

However, the apparent determinacy of the modelled system may be emphasised, and this may minimise the impact of irreducible uncertainties. In part, this may reflect a belief that models are able (at least to some extent) to overcome such indeterminacy and find the world to be more deterministic at some notional deeper level:

**E2 (Scientific paper)**

Our confidence in the model goodness of fit is reinforced by the extent to which the simulation model parameterised using the inference model parameter estimates was able not just to reproduce the pattern of the 2001 outbreak, but in many cases predict exactly which farms would become infected. …While the timing of the infection of individual farms can be much less well predicted, the level of ‘determinism’ in the epidemic process our analysis has revealed may make detailed real-time prediction more feasible. Indeed, given real-time prediction conditions on the current state of an epidemic rather than the state at some early selected time point, one might expect to do rather better than the degree of correspondence seen here between observations and data, especially later in the epidemic. [27].

This account adopts a more empiricist repertoire than that of E1 and implies that the epidemics are, in key ways, deterministic at the individual (farm) level. It thus implies that the key cause of the apparent indeterminacy is a failure adequately to measure necessary parameters.

The above extract (E2) from a scientific paper describing a particular model contrasts markedly with the following extract from the final report of a group set up to review Defra's use of modelling, completed the following year. Of key interest is that one of the authors of this report was also an author of the previously quoted paper:

**E3 (Report)**

…models of livestock disease dynamics may include every farm in the UK, but model output at the farm-level has such high levels of uncertainty that the results at that scale are of limited utility, meaning aggregated output is usually used. [28].

Part of this apparent inconsistency lies in the inevitable ambiguity in expectations of future model improvement, and the confusion sometimes observed between expected model performance, and promised or aspired-to future performance (e.g. [29]), and actual current performance. Examination of these extracts (E1, E2 and E3) highlights variable interpretation of the potential for models to predict. This variation emerges through the differential application of elements of the empiricist and contingent repertoires. Of note is the different intended audience and purpose of the extract documents: a textbook for modellers describing the science of modelling (E1); a scientific paper providing evidence to support application of a new method in epidemic situations (E2); and an internal report to a government department scrutinizing issues surrounding use of models in policy-making (E3).

Emphasis on the determinacy of a model may ultimately lead to expression of model results as predictive truths [30]. For example, the following statement, relating to the models used in the 2001 FMD epidemic, suggests the model output provides an exact guide as to the events that will occur under a range of scenarios, with no allowance for any form of uncertainty:

**E4 (Parliamentary Inquiry)**

And, of course, what it stresses is the importance of the scientific modelling that was done to project forward from any point during the outbreak as *to what the outcome would be* given various control scenarios. [italics added] UK Chief Scientific Advisor, Prof David King, quoted from [31].

King's statement, made to the UK Parliamentary Inquiry into the impact of the 2001 FMD epidemic, illustrates the use of strong program rhetoric (hard sell [16]). Here, modelling science unequivocally indicates the different futures open to decision makers; no accommodation is made for contingency, nor thus for alternative scientific views. This contrasts with the weak program rhetoric (soft sell) adopted in a scientific journal paper read predominantly by microbiologists:

**E5 (Scientific paper)**

The model [i.e. as referred to by King, above] *can* be used to *explore* the expected impact of alternative control strategies. [emphasis added] [32].

This soft sell presents modelling as an exploratory tool to assess what might be of the impact of alternative choices, implying that modelling provides a starting point for discussion and need not dogmatically warrant a specific action in response.

### Production of models

A range of technical practices form the overt tools through which a model is formulated, implemented and tested, broadly those that define and design the model, and those used to test or validate the model. To explore discourses of definition and design we draw on three activities: selection of input data, parameterisation and enumeration of uncertainties. In the next section we explore the discourse of model testing.

Some models of the UK 2001 FMD epidemic utilised input data of almost unprecedented detail. For example databases, developed for a range of purposes, contained information on farms, including their location and the species of livestock present on these farms. As farms have fixed locations (although this is contested as, for example, some farms exist as multiple, disconnected geographic locations yet would appear as a single location in a database [33]) and the spatial pattern of the epidemic was used to infer the predominance of short-range transmission, incorporation in models of location data was seen by many as important. However, accounts of the quality of these data vary widely.

**E6 (Scientific paper)**

The epidemic has generated a unique data set describing the spatial spread of an infectious disease between fixed nodes, i.e., livestock farms. This, together with the availability of data on the location and livestock composition for all UK farms [collected by the Department of the Environment, Food and Rural Affairs (DEFRA)], offers an unusual opportunity to explore the impact of spatial and individual heterogeneities on the course of an epidemic and the importance of these variables for the design of appropriate disease control programs [22].

**E7 (Interview – modeller)**

Yes, but you know, the people who curate the database of farms really don't care where they are. You know, why they've got that information in they probably don't know. They've got an address of the person they write to, that's the only real spatial location they need and the fact that the geographical co-ordinates place the farm in the middle of the North Sea you know, so what. And they probably work on a 5% acceptable accuracy you know, error rate anyway or some kind of error rate and they're not going to spend a huge amount of resource to make sure that the x, y co-ordinates are actually spot on.

The description of the data presented in the scientific paper (E6) is short and impersonal; it gives logical precedence to the existing data which, by implication, represent the relevant aspects of the natural world. In contrast the interview (E7) gives a more informal account of the role of individuals, organisational culture and resources in the production of the data and their effects on data quality. We see a stark contrast between the empiricist and contingent repertoires, respectively, and of the effect of these discourses on the production of an account of the uncertainty present in these data, and in the model.

Singleton [17] describes the functional role that instability can have in stabilising a technology. Extract E7 (above) highlights uncertainties in essential data, which may be interpreted as undermining, to an unknown extent, results derived from models using such data. However, as the view from a different modeller below indicates, this same instability (data uncertainty) is emblematic of the benefits of modelling.

**E8 (Interview – modeller)**

[Where] modelling, in its broader sense, is becoming more capable of making a difference is in what's, kind of, generally called data synthesis or taking data from a noisy and incomplete data from a wide variety of sources and pulling it together to make something which is a little bit more than the sum of its parts.

Here, the very issue that seems to undermine models in E7 is seen in E8 as strengthening their role, which is represented as being able to accommodate uncertainty and ambiguity, by synthesising disparate data from disparate contexts.

Parameters are numerical characteristics that, as a set, define the behaviour of a model. For example, relatively simple models may be defined with just a few parameters defining the ‘birth’ and ‘death’ rates, the duration of infectiousness and the rate at which infectious individuals transmit infection to susceptible individuals. Estimation of these parameters (an activity referred to as *parameterisation*) may take a range of forms. Pre-existing information may be used, particularly for those parameters believed to be observable. For example, information obtained from study of infected individuals may be used to infer some values, such as the duration of infectiousness. Other parameters are not observable and hence must be estimated in some way. In the 2001 UK FMD epidemic key parameters to be estimated included those that determined the transmission of infection from infected to uninfected farms.

**E9 (scientific paper)**

**Figure 1:**
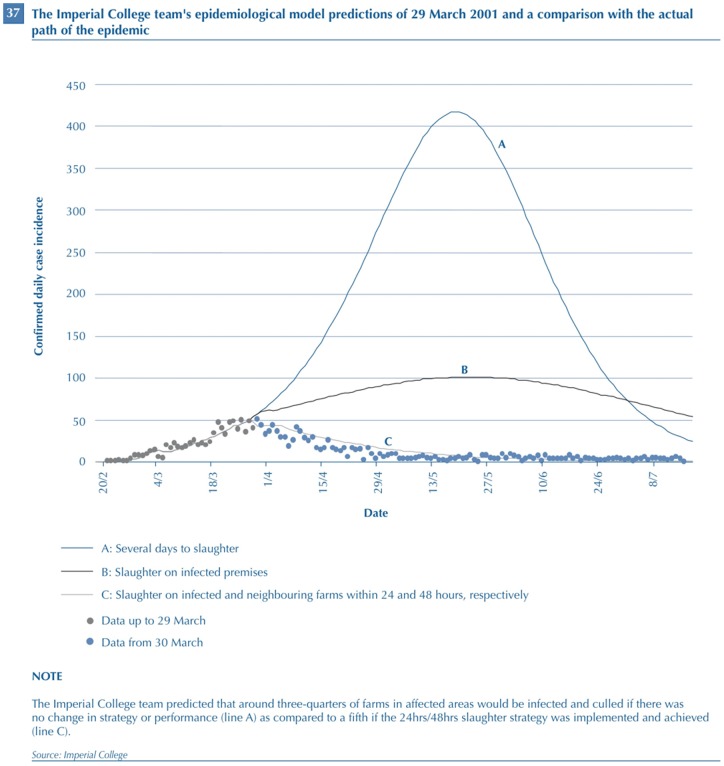
Graphical representation of predictions, made by Imperial College's modelling team, of the UK 2001 foot-and-mouth disease epidemic based on data up to 29 March 2001, and comparison with the subsequent epidemic data. Published with permission Imperial College, London, and the National Audit Office. This figure originally appeared in National Audit Office Report ‘The 2001 outbreak of foot and mouth disease’ [18].

A key modeling decision is how to represent the local and regional spatial clustering of FMD cases (Fig. 1A), which precludes the use of standard models based on homogeneously mixed host populations (1). This contagion is quantified by the spatial infection kernel of the disease (2) (Fig. 1B); after the introduction of movement restrictions in late February, the kernel shows a high probability of local spread, with a tail of less frequent longer range “sparks” of infection. Some of the local effects caused by the clustering of infection can be modeled implicitly, with deterministic approximations (3, 4). However, to explore the full spatiotemporal dynamics of the epidemic–in particular, the highly irregular behavior in the epidemic tail–we use a stochastic, spatial, individual farm–based model. The stochastic nature of transmission generates inherent uncertainty in the ability to predict events; however, in this epidemic, there are also two more systematic sources of uncertainty. First, we only have a qualitative grasp of the multifaceted nature of FMD transmission between farms (5–8); key transmission parameters must therefore be derived by fitting the model to the epidemic data. Second, there are biases and various lacunae in the epidemiological and management data used to construct the model (9). We summarize how these uncertainties affect our predictions in the supplementary material (10)…

…Because of the rapid transmission of the virus between livestock in the same farm, it is reasonable to treat the farm as the individual unit (4, 11–13), classifying each holding as either susceptible, incubating, infectious, or slaughtered. We also incorporate the heterogeneity in farm size and species composition (13) by allowing the susceptibility and infectiousness of farms to vary with the type and number of livestock (14). In principle, the necessary parameters can be estimated from the observed pattern of cases by maximum likelihood. However, we cannot rely only on this, because of spatial and temporal biases in the data (9). We therefore adopt a two-stage approach, generating an initial fit by maximum likelihood, then refining it by least squares fits to regional epidemics (10) [22].

**E10 (Interview – modeller)**

In terms of the actual pathogen itself, yes, the amount of data actually, which was useful to modelling, on the transmission characteristics of the disease, even the natural history of the disease within infected animals, was rather limited. I mean, effectively most of the – well it's data or knowledge out there was qualitative rather than quantitative and so, I mean, what we tended to find was that a relatively small group of scientists worldwide had been working on foot and mouth. They were the experts called upon historically to advise and control of the epidemic and they, sort of, had a, kind of, gut feeling of this is how it behaved and a lot of it wasn't really quantified in any, sort of, rigorous way.

[...]

I mean, one could piece together reasonable estimates, but a lot of things weren't available. That has changed to some degree, so there has been a recognition since that point in time that some of the things which were being focused on perhaps were less relevant and other things were being focused on more in terms of – particularly some of the transmission studies undertaken, but also some of the basic virology.

*Interviewer: So how did you cope with that when you were trying to present results…?*

I mean, eventually you present a set of assumptions. I mean, to that sort of group, it depends on the context, but effectively you present a set of assumptions about things like incubation periods relationship between infectiousness and level of symptoms, how long it takes symptoms to clear, those sort of things to a, kind of, expert group and they comment on whether they think they're reasonable.

A feature, clearly evident in the first part of scientific paper E9, is the repeated posing and resolution of problems, a practice referred to as deproblematisation by Singleton (1998). We see that standard models with homogeneous mixing are precluded, so a spatial infection kernel is introduced; the stochastic nature of transmission can be incorporated using a stochastic model; derivation of transmission parameters using data avoids problems due to limited existing information. Although the extract opens with a statement highlighting that a decision must be made, the authors are merely impartial actors in this decision-making process, taking actions as required in a purely technical fashion. The use of an empiricist repertoire here is obvious. For example, in the second sentence, the “contagion is *quantified* by *the* spatial infection kernel *of the disease*” [our emphasis]: there is no sense that this kernel is being estimated in a fashion that requires the judgement of individuals; the authors are working with *the* kernel, rather than one of a number of possible variants, and; this is the kernel of this disease –a property of the disease, not a product of scientific activity. Furthermore, where there is recognition of the contingent nature of knowledge about FMD there is an empiricist solution which “must” (and, presumably, can) be undertaken “fitting the model to the epidemic data”. The existence of biases and lacunae is mentioned, but further information regarding these is relegated to the supplementary material. Access to this information requires reference to endnotes and, from there to online documentation (further discussed below.) This extract emphasises the power of modelling to deal with what may otherwise be substantial problems in interpretation of the outbreak data.

In contrast, in interview E10 we observe a strongly contingent repertoire where a very different process is outlined, one highly subject to a wide range of influences. Both scientific paper E9 and interview E10 are concerned with what is basically the same parameterisation process, but they are from different sources and each is describing the actions of different groups of people. Hence, some of the differences are likely to arise due to the different events being described. Nevertheless, each takes a very different perspective – one largely technical and deterministic, the other presents a process that is informal, intuitive and contingent.

Similar issues to those voiced by modellers in interviews E7 and E10 are used by critics of modelling to refute the ability of models (or a particular model) to inform action.

**E11 (Review paper)**

The 2001 predictive models were constructed in an environment of poor-quality data (e.g. they used out-of-date census data for stock levels), and poor epidemiological knowledge (e.g. the transmission characteristics of the virus strain, and the distribution of the initially infected farms, were unknown). Therefore, their use as predictive tools was inappropriate [34].

However, note that in this review the empiricist repertoire is reinstated in the assertion of the primacy of data, and is juxtaposed with a more contingent repertoire when the role of modellers as actors in the modelling process is highlighted in their decision to use “out-of-date” data. This combination of contingent and empiricist repertoire creates a dissonance that has the effect of undermining modelling as an empiricist activity. Here we see that what was stated to be “data on the location and livestock composition for all UK farms [collected by the Department of the Environment, Food and Rural Affairs (DEFRA)]” (Scientific paper E6) becomes “poor-quality data” and “out-of-date census data for stock levels”, whilst “a unique data set describing the spatial spread of an infectious disease between fixed nodes, i.e., livestock farms” (scientific paper E6) and transmission “quantified by the spatial infection kernel of the disease” (scientific paper E9) become, simply, unknown. In fact, the reference to farm location and composition data made in the main text of scientific paper E6 is expanded in the online supplementary material (E12) to reveal that these data are indeed sufficiently incorrect to undermine some aspects of the model's validity. (However, note that each issue raised is immediately deproblematised).

**E12 (Scientific paper)**

The presence of biases in the livestock data is well accepted, and probably leads to a significant over-estimate of the number of sheep in Wales at the start of the epidemic and smaller variation in other areas. This effects [sic] our ability to model the exact spatial distribution of cases, which may explain why we slightly overestimate the number of cases in Wales and Yorkshire. Such a bias will also alter the proportion of mixed and single-species farms recorded in the database, although not sufficient to change our quantitative conclusions. Some of this bias will be absorbed into the parameters by the fitting procedure. (Online supplementary material to [22]).

These variable interpretations of information used in models reflect the battle between those supporting and those refuting their legitimacy as predictors of the effect of, for example, interventions. Comparison of these texts emphasises the contested nature of the calculus of uncertainty and that utilising the ‘all models are wrong, but some are useful’ mantra to endorse the use of models is problematic.

The proliferation of potential sources of uncertainty that arise as models move from a conceptualised to a contextualised system and thence to a structured model, makes enumeration of all of these uncertainties an impossibility, let alone full consideration of all their implications for the veracity of the model conclusions. Hence, worthwhile recommendations such as: “In a case when an assumption simplifies or approximates the underlying epidemiology, it should be clearly stated why this assumption has been made and how this may influence the results” [14] can, at best, be only partially fulfilled and many potential uncertainties must remain unstated and perhaps unconsidered. This effect may be accentuated within the empiricist repertoire because technical efforts and subsequent discussion tend to focus only on reducible uncertainties, for example highlighting the need for more precise measurement.

As mentioned above, Keeling *et al* [22] enumerate selected uncertainties present in their model, very much in keeping with recommendations for best practice [14]. It is noteworthy that, although briefly mentioned in the main text, this list is found only in the accompanying online supplementary material, potentially inhibiting its accessibility. In all, seven kinds of uncertainty are listed, although each could be viewed as a composite of many underlying uncertainties. Here we present only the first of these.

**E13 (Scientific paper)**

*Relative infectivity and susceptibility of sheep and cattle.* Experimental results agree with the pattern of species differences used within the model. Quantitative changes to the species parameters will modify the predicted spatio-temporal distribution of outbreaks; our parameters have been chosen to give the best match to the location of high risk areas. However this choice of parameters is contingent on the accuracy of the census distribution of animals on farms [22].

This uncertainty actually encompasses at least four underlying uncertainties (about each species and each process), but could also include uncertainties about potential variation in each process (for example relating to age, breed and other factors that, although poorly described, may influence individual animals' immune systems). Hence, this single listed uncertainty embodies a wide range of independent potential sources of uncertainty, although most are not mentioned or discussed. As is well understood, but may need repeating here, these are not only additive in their potential cumulative error-magnitudes, but may be multiplicative. Hence, authors must decide what to present, what to leave out, what to prioritise and, ultimately, what is to be achieved through the communication processes in which the model becomes an actor. The representation of uncertainties through the production of the model is, therefore, contingent on the actions of the authors, and alternative choices and representations are possible. Importantly, omission of any specific uncertainty in communication of model results may prevent or limit its consideration by consumers/users of the model results. We contend that selective omission, reinforced by an empiricist repertoire, is a recurrent feature of formal modelling discourse, and that this asymmetric accounting results in understatement of the potential implications of uncertainties in what are usually mixed biological and social systems, even when these are recognised by modellers in less formal discourse.

### Testing models

Testing is a vital part of the production of a model. Although often referred to as a distinct activity (as we do here), which chronologically follows construction of the model, in practice these activities are often closely iterative, with early forms of a model undergoing revision based on testing until a final version is accepted. Hence, more complex models may be created incrementally by adding detail to simpler models, which are tested at each stage.

While technical testing methods represent a key part of the modeller's armoury, the lack of consistent interpretation of necessary standards and procedures for verification, validation and parameterisation is recognised [28] and this introduces new uncertainties regarding the veracity and legitimacy – thus also the public policy authority – of the conclusions based on these approaches. The practice of testing may include activities defined as model verification and validation, sensitivity analysis and scenario analysis. Here we focus on validation.

Model validation has multiple definitions. It has variously been described as the “process of ensuring the model is an adequate representation of the physical or biological system being represented” [28]; or more loosely as ensuring “that a model is acceptable for its intended use because it meets specified performance requirements” [14]; or, more restrictively, as “checking the model outputs against independent data sets” [35], and more definitions exist. Of note in these definitions is the use of qualities such as “adequate” and “acceptable”, which must be subjectively applied. Even apparently purely scientific norms such as “specified performance requirements” can only be defined relative to what are social definitions of appropriate uses of such models.

Woolhouse *et al* [14] define three kinds of model validation. The first is the restrictive definition used above, that of comparison against independent data. The second involves recreation by the model of the key features of the input data, whilst the third involves comparison with outputs of other models. We draw on our data to consider each of these approaches in more detail.

#### Comparison against independent data

It is well recognised that in the case of many infectious diseases, particularly epidemics, comparison with independent data is frequently not achievable, due to lack of additional, comparable events [14], [28]. However, early in the 2001 FMD epidemic, models were used to make predictions of its future course, and these predictions were compared with subsequent observation. Illustrations of these predictions, such as those in Figure 1 (reproduced from [18]) appear very impressive. The models suggested the benefits of introduction of more extensive culling to include farms neighbouring infected farms, and to do this quickly (i.e. within 24 hours of diagnosis on infected farms and within 48 hours of that diagnosis on neighbouring farms: the so called 24/48 rule, or ‘contiguous cull’).

The following extract from the UK Parliamentary enquiry [31] refers to a figure similar to Figure 1.

**E14 (Parliamentary enquiry)**

Professor King: It was not just a computer model, these models were learning from the way this outbreak happened. Please do not say it is just a computer model. It was picking up on incubation periods, and so on, from the early stages of this outbreak. Without that, we would have been modelling any sort of outbreak; it was this outbreak that was being modelled. And when we give these figures, like 17 per cent of contiguous farms and the argument for the contiguous cull, it is all based on this outbreak, and when it was out of control we were saying, “This is how you will bring it under control.” And what I would like you to do is to look at the very impressive figures; if you compare Figure [1], which is the predictions that were made, the curves A, B, C, with [the epidemic data – blue dots], which is how the epidemic developed, I think you have got to agree that that was not bad agreement, the prediction was not too bad.

The line C in the figure, which was generated from a model based on the epidemic data represented by the grey dots, does indeed appear to follow closely the pattern of the subsequent outbreak (represented by the blue dots). King (then the UK Government Chief Scientific Adviser) adopts an empiricist repertoire and strong program rhetoric to sell the authority of the model in decision-making. Data are given priority in the production of the model, which ensures that these are not the results of just any model, but rather are obtained from a very specific model that encapsulated the important features of the real outbreak. There is a clear implication that the model results objectively enforce a particular course of action (represented by line C) and that the data observed subsequent to the adoption of this action validate the model, this view being repeated elsewhere (e.g. [32]). However, alternate readings of the data underpinning the figure are available.

**E15 (Review paper)**

What caused the 2001 epidemic to end? This is likely to have varied between regions. Reducing the period of time before an animal is slaughtered and increasing detection rates no doubt contributed to the decline of the epidemic, and the revised policy measures were designed to facilitate this. However, reconstructions of the epidemic indicate that the rate at which new infections were arising peaked between 19 March and 21 March, and the number of reported cases peaked on 26 March – before these new policy measures were implemented. Therefore, the switch to more stringent control procedures could not have been responsible for this initial reduction.

…

Given uncertainties in the data and the reliance of these models on assumptions that are necessarily crude and also difficult to verify, it is difficult to make the argument that mathematical models showed that implementation of widespread and intensive culling was the only tenable option. Models did show clearly, and at a relatively early stage, that a traditional policy, as previously implemented, was not sufficient to prevent the development of a very large epidemic. However, the main arguments in favour of a CP [contiguous premises] cull are simpler decision-making and ease of management, together with the benefit that, in a time of great chaos and uncertainty, a clearly defined policy with simple goals can be of both logistical and political value. [36].

The potential that the reduction in cases occurred prior to changes in policy raises doubts regarding claims made for the link between model-predictions and the observed data. In this passage, multiple contingent details are revealed. Firstly, the data are reconfigured as uncertain and the model assumptions as crude. Interpretation of the model results is then revealed to be a subject for argument rather than a self-evident empirical phenomenon. The authors also undermine assertions implicit in the previous quote from King that the control options recommended were a biological imperative, but rather suggest that much of the benefit of the control measures adopted was politically and managerially derived. Hence, even the more rigorous form of validation, where a model is tested against independent data, can be contested and result in flexible interpretation.

#### Comparison of model output with input data

In practice, such independent data is rarely available and observed epidemic data may need to be used for both model development and for comparison with model output as part of the process of validation (the second form of validation noted above). However, this method is seen as not providing true validation [14]. During model production input data can be used to guide the model structure and to estimate the model parameters, and input data may even be altered in order that model output reflect the observed events. For example, input data for UK FMD models have been adjusted in order to achieve good fit with the observed data [37]. This adjustment, sometimes called ‘parameter-tuning’, is not a trivial process and it may be expected that it would impact the way the model represents knowledge of the underlying system. Some models ‘tuned’ regionally varying parameters in order to ensure the average simulation results match the observed data [38], whilst others modified the input demographics (by reducing the sheep density in particular regions) [22], [39].

The expectation that model output should recreate key features of observed events highlights two additional important points. First, the assumption that a good fit between model output and observed events is evidence of a good model, requires reasoning that cannot be used to demonstrate the validity of a model. There may be many models which provide a good fit to the observed data; in which case the model in question is just one of these. Further, the body of models that satisfy the observed data may provide very different predictions when confronted with new initial data or new modifications to reflect different interventions [40].

Secondly, it is worth reconsidering the supposition that an appropriate model should generate results that match the observed events. This belief assumes that any observed epidemic event is very similar to the average of all potential realisations of this event in the underlying (stochastic) system. However, the average is a special case, and in the majority of instances the observed event may be expected to differ from the average by an unknown amount. For example, modelling of the UK 2001 FMD epidemic typically assumes that the observed epidemic was the average epidemic that could have been expected [21], [22], [37], despite being one of the two largest epidemics of FMD ever recorded in the UK [41]. When an observed epidemic is different to the average of many (hypothetical) occurrences of an epidemic with the same dynamic properties in an identical system, model-parameters estimated from the observed epidemic data will differ from their ‘true’ values. This effect was likely the cause of the failure to predict accurately numerous parameters in a study using data from a single simulation of an outbreak of avian influenza, though this was not stated as a probable cause by the authors [42]. Further, if, as Jewell *et al* (2009) state, key parameters are peculiar to individual outbreaks, modellers of these events will *always* be confronted with a single (perhaps partially observed) epidemic from which to estimate parameter values or distributions, yet will be uncertain as to whether the observed epidemic is typical or large or small for the true underlying parameter set. Although parameters calculated in this way (from observed outbreak data) may enable the model results to fit the observed data ‘well’, assessment of the effect of control strategies may clearly be misleading if their impact is modelled through modification of mis-specified parameters.

Despite these concerns, comparison of observed epidemic data with model output is used to assess the validity of models, and this is often mistakenly treated as an unproblematic process.

**E16 (Scientific paper)**

There is very good overall agreement between the average of the model replicates and the reported cases (Fig. 1). The observed qualitative pattern of variability is also captured by the simulations–note, though, that we do not include day-to-day environmental stochasticity in the model. The average of our simulations slightly underestimates the epidemic, after the decline in early April. The first part of this is probably due to over reporting of cases (10). We may also slightly underestimate the latter stages of the epidemic, probably because of small systematic secular changes in transmission not currently included in the model, such as the mid-May turnout of dairy cattle from winter housing onto pasture.

The high degree of spatial correspondence between model results and data depends on the inclusion of species and herd-size heterogeneities in transmission (10). The model captures the main regional foci of infection in Cumbria and Devon, although there are some departures that may be attributable to biases in the data (9) or local heterogeneities. Rigorous statistical assessment of the spatial fit is complicated by farm-level variation between simulations. The numerical simulations from 23 February to August capture the overall shape of the epidemic. Although this is not an independent comparison (because the parameters are estimated from the fit), the model's ability to capture the shape, spatial distribution, and variability of the epidemic is encouraging [22].

Although employing empiricist repertoire these test results are actually presented in very imprecise, contingent terms. Pinch et al [16] observed discourse surrounding the testing of a very different technology (clinical budgeting). Similar to their study, we observe above that the results of the validity test are presented as a qualitative interpretation of a figure (i.e., in the terms of Pinch *et al* [16] evaluation of the results is based on weak programme rhetoric). There is no attempt to delimit the conditions under which this test could be falsified. Furthermore, again in agreement with Pinch et al [16], we see that any ‘failure’ of the test (here indicated by lack of agreement between predicted and observed events) is ascribed to the particularities of the environment (such as those that may be due to under-reporting of cases, turnout of cattle, biases in the data or local heterogeneities) whereas areas of agreement are assumed to be evidence of success.

#### Comparison of different models

The third method of validation, comparison of model results with results of separate models, is frequently used to support the validity of a model or of its policy-related conclusion of the model (e.g. [26], [32]).

**E17 (Scientific paper)**

Based on data collected during the epidemic, prospective modelling using a variety of approaches gave the same conclusions: (i) that the epidemic had not been brought under control by ‘traditional’ methods, and (ii) that neighbourhood control measures (the contiguous cull) could bring the epidemic under control and result in a net saving of livestock. Retrospective analyses suggest that the subsequent course of the epidemic was consistent with a beneficial impact of the contiguous cull and that it would have been difficult to achieve a better outcome using reactive vaccination, which would have required very large-scale vaccination programmes to have been implemented quickly [32].

Here, the test used to confer validity to the models relies on comparison of the model with regard to two quite general conclusions. The first was that the epidemic was not under control, and hence that ‘traditional’ methods had failed (and, presumably, that non-traditional approaches were now needed). The second general conclusion was that culling needed to be extended to include neighbouring farms, and that this would result, ultimately, in fewer animals being killed. Again, the validation tests utilise results expressed in weak program rhetoric, and without information on whether agreement was also present for other relevant model-generated conclusions. In fact, rather than seeking direct validation of the models themselves, the primary function of the above statement is validation of the *conclusions* of those models. The statement does not require any consideration of the technical qualities of the models or even why particular decisions were made – the key point being tacitly negotiated is that the correct policy action was taken. Hence, the form of validation above eliminates issues of uncertainty in production and thus validity of the models from the discourse.

Returning to more general consideration of this form of validation, it is frequently stated that similarity of results provides, at best, only weak evidence of model validity. This arises because there may be tendency toward consensus due to similarity in, for example, input data, parameter values, model construction and/or the conceptualisation and contextualisation of the system. This concern has been raised regarding UK 2001 FMD modelling practices, which all shared “certain fundamental similarities” [36] and were also noted in interviews with modellers.

**E18 (Interview – modeller)**

I mean, there's people working on the … modelling now who are all, kind of, competing institutions and individuals at one level, but they're all working together harmonistically…the trouble is that these – the groups don't tend to operate in that much isolation and there's no rogue group.

**E19 (Interview – modeller)**

This is going back to the notion of, to what extent are the models actually different from each other? But if, in many respects, the models are actually the same as each other, then the range of predictions they generate may not be wide enough, 'cause they may all share the same inaccuracy.

Similar concerns were raised in the final report of Defra's Science Advisory Council [28], set up to investigate how Defra uses modelling, and which encompassed and compared modelling of infectious diseases, climate and air-pollution. Interestingly, in this report this concern was raised only for climate models and not explicitly extended to other modelling activities such as infectious diseases. [28].

Finally, as noted by Haydon et al [36], all models used in the UK 2001 FMD epidemic considered a narrow range of policy options and “it is difficult to make the argument that mathematical models showed that implementation of widespread and intensive culling was the only tenable option.” Therefore, the strong program rhetoric adopted in the comparison of models (and the inferred validation of these and/or their conclusions) masks the weak program test that is used, ignores issues that may bias the models toward developing similar mutually affirming conclusions, and limits the scope of discussion by disregarding alternative potential policies.

### Negotiation of uncertainty and the authority of modelling

Many modellers we interviewed expressed awareness of complex issues associated with communication of uncertainty to decision-makers, with modellers being simultaneously aware of the scientific basis of the models and the need to safeguard the influence of modelling as a tool in decision-making.

**E20 (Interview – modeller)**

It's very dangerous to say you don't believe this model before you start. It's quite a hard trick to pull off to convince the policymaker that the model has value and should be believed and they should base their policy on it and at the same time explain that actually the model, it's not true, is wrong.

**E21 (Interview – modeller)**

If the modellers believe a model's giving important advice, even though there's a level of uncertainty in that advice, how – it's a real issue – how hard should you – if you feel the advice is good advice and important, how hard should you push it? ‘Cause if you’re – if you take a very open approach, saying, “Well, this may or may not be correct, there are different possibilities,” then you run the risk that what you consider important advice may be ignored.

These modellers express the concern that exposure of their true understanding of the uncertainties of a model would undermine its credibility and prevent its effective contribution to decision-making. This belief articulates two perceived distinctions between the modellers and the model-users. The first is that modellers are more aware of the uncertainties of the model than are model-users. The second is that, despite a detailed appreciation of the uncertainties in a model, modellers remain confident that the model can inform policy, while in contrast, it is only through (partial) ignorance that model-users can employ the findings of models in decision-making.

This difference in knowledge about uncertainty accords with a further key insight from social studies of science: MacKenzie's ‘Certainty Trough’ [43]. In his book detailing the ways in which missile accuracy is produced and understood, MacKenzie shows that those closest to the production of knowledge (in our case the model creators) are most aware of its uncertainties, whereas those further distanced from model creation (model-users) may be less aware, as they were not exposed to the complex contingencies of production which, as we have shown, are essential to modelling. The greater the distance from model construction therefore, the more diminished are the chances of understanding the contingent labours and knowledges known to specialists [44]. Hence, whilst model-users may have some appreciation of general issues relating to uncertainty in models, they may be poorly placed to judge the depth or impact of these. This difficulty was articulated by one respondent, quoting a colleague who had expressed frustration with a model: “Everyone knew it was crap, but nobody knew why”. The model-users we interviewed expressed varying degrees of sensitivity to uncertainty, and flexibility in their interpretation of the importance of uncertainty. For example, for one respondent the responsibility for model results to be useful rests with the modellers.

**E22 (Interview – model-user)**

…I don't spend too much time thinking how uncertain are the parameters of this model? So at that level the representation that's been made of the disease system I accept that, once it's got to me, some sort of scientists have done some verification of it and it's now believable and can be used as a reasonably sensible representation of the way the next 1,000 years of FMD outbreaks may turn out to be …

However, reliance on modellers' interpretation of uncertainty may be problematic: modellers have a deep professional and emotional investment in their work. As highlighted by Lahsen [45], investigating the case of global climate change modelling, knowledge producers are “certainly not critics” of their own technology. The flexibility with which issues of uncertainty can be represented by modellers, as already illustrated in this paper, makes any characterisation of uncertainty partial and amenable to selective presentation based on strategic choices [46]. Furthermore, Shackley and Wynne [46] contend that oscillation between strong, empirical claims to scientific authority (models as predictors) and more modest, contingent claims about models as aids to understanding and discussion (models as ‘heuristics’) serves to preserve certainty, and that modellers present and encourage interpretations of models as ‘truth machines’ in discourse with model-users (and other external audiences) in order to preserve the authority of their models.

It is therefore interesting in our case, (in contrast to that of MacKenzie) that some model-users expressed a desire to have a more detailed appreciation of potential uncertainties. However, this aspiration may be tempered by a lack of necessary technical expertise to assess all implications of a model's uncertainty. As noted by one model-user, there was a desire for some level of understanding of uncertainty:

**E23 (Interview – model-user)**

I want to understand some of it. Clearly, what I want to know is the inputs in broad terms, so therefore what are the datasets that are being used? Are you satisfied with the quality of them? Have we got satis – are we satis – can we get assurances about the quality, timeliness and accuracy of the data? I want the modelling team to understand the disease in the broadest sense of it. I don't want people to make assumptions about disease.

This extract exposes a limited wish list that would not be able to expose all uncertainties. There is no sense of a formal consideration of uncertainty, but rather only a need for assurances regarding the input data and for the modellers to “understand the disease in the broadest sense” and to not make assumptions. It is of interest that the speaker begins by seeking to be satisfied with the quality of the data, perhaps implying an active examination of these data-sources by the model-users, but immediately hesitates before settling for assurances of quality. Although not specified, this appears to place the responsibility for quality-testing onto another party, probably the modellers themselves. This respondent also requires that the modellers do not make assumptions – yet modellers themselves recognize that models are, by necessity, simplifications of reality which require assumptions to be made.

Other model-users recognised that the impact of uncertainty on decision-making was context dependent. For example, if a model is to be used in support of pre-existent policy-decisions and the model results are compatible with that decision, then the model has little impact on the decision, it merely provides some additional support for the decision-maker.

**E24 (Interview – model-user)**

If you need some information to make a decision, you want to say that the evidence is backing your decision, you might have already made a political decision or a decision based on perhaps where you know the industry is happy to go and you're saying, “Oh, well, in order to tick this box that says, yes, I've had evidence or I've commissioned some work that says, yes, there's evidence that supports our approach,” then you're less interested in perhaps what that uncertainty is.

Here the decision has been made and, provided the model supports that decision, model results may be used almost irrespective of issues of uncertainty because the model is actually having little influence on the process of decision-making. However, when a contrary situation was postulated (that is, a decision is made that is contrary to the conclusions of a model) uncertainty was usually not proposed as the cause of that decision, but rather this was attributed to the role of other sources of knowledge.

**E25 (Interview – model-user)**

I think it's more about perhaps, you know, perhaps if you've been doing a piece of modelling work and it says that you should do this, because a decision has been made not to do that it's not necessarily because the evidence has not been listened to but the evidence is only part of that decision making process.

Our general conclusion was that model-users felt ill placed to judge a model on its technical merits and relied on interpretation of model uncertainties by modellers themselves. This places modellers in a strong negotiating position, which users typically counter, if needed, by reference to external factors or sensitivities.

Furthermore, discussion of the role of modelling in decision-making may be used more generally to modify interpretation of the impact of uncertainty. For example, the role of a model in the decision making process may be de-emphasized by highlighting other factors important in this process and/or highlighting that the model merely supported an existing decision. Such behaviours were evident in our data, for example one modeller here referring to models of the 2001 UK FMD epidemic:

**E26 (Interview – modeller)**

Yeah, so, my perception of it was that the modellers were generally – and the models – were generally used to bolster and reinforce the decisions that had already been made about how to handle it, rather than directing anything new or novel.

Hence, in this interpretation the model is, perhaps, a relatively minor part of the information used to make or support a decision, rather than the basis of that decision.

In contrast, critics of the role of models during the UK 2001 FMD epidemic [33], [47]) emphasize the central role assumed by models in the decision making process. For example, the very titles of publications criticizing the role of modelling stress a central role for modelling in decision-making; “‘Carnage by computer’: the blackboard economics of the 2001 Foot and Mouth epidemic” [47] infers a direct link between the modelling activities undertaken with computers and the large scale culling of livestock, whilst “Destructive tension: mathematics versus experience – the progress and control of the 2001 foot and mouth disease epidemic in Great Britain” [33] suggests that modelling trumped more experience-based perspectives in development of control policies. By ascribing a definitive role for the models in decision-making, combined with detailed exposition of possible uncertainties, critics of the use of modelling construct a perspective in which models are not able to act reliably in decision-making.

Thus, whilst the notion of *wrong but useful* assumes that the *quality* of a model can be ascertained in order to decide whether or not the model should be ‘used’, in practice issues of model-quality may be ignored, asymmetrically applied and superseded by additional factors. We contend that rather than being based predominantly on beliefs about quality, the usefulness and authority of a model may at times be primarily based on its functional status within the broad social and political environment in which it acts.

## Conclusions

We have illustrated the flexibility with which issues of uncertainty are managed in the discourse of infectious disease modelling. We have highlighted the occurrence of both empiricist and contingent repertoires [15] and different accounts of uncertainty provided through these repertoires. We have also found asymmetric accounting of evidence and uncertainties in modelling and its uses, and contend that this may be a more general form of the asymmetric accounting for error identified by Gilbert and Mulkay [15].

Our analyses have also identified the use of strong and weak program rhetoric [16] on the significance of uncertainties and the truth-value of models, and, particularly, the way these may work to support the role of modelling through testing. Finally, we have found evidence to suggest that identification of uncertainties, combined with their ‘deproblematisation’ can act to stabilise the role of scientific modelling in decision-making, in the same way as that identified by Singleton [17] for the role of laboratories in the UK cervical screening programme.

We have also explored the role of negotiation of uncertainty in the development of authority of models to inform decision-making. We found that modellers, when communicating their results to users, identified a tension between being open regarding issues of uncertainty and the need to protect the authority of modelling by minimizing the impact of that uncertainty. We also found that awareness of and expression of certainty in our study were in keeping with MacKenzie's ‘Certainty Trough’ and that policy-makers found issues of uncertainty, and its recognition, problematic and employed different ways of managing this. We also found that the need to assess the impact of uncertainty was dependent on the role of the model in the decision making process.

Our analysis prompts three main conclusions: (i) discourse around dynamical disease modelling has many similarities with that found in other scientific and technological practices, (ii) the flexible discourse of uncertainty in infectious disease modelling renders uncertainty a plastic quality which is amenable to ‘interpretive flexibility’ and negotiation, and (iii) the utility of models in the face of uncertainty is a function of this flexibility, the negotiation this allows, and the contexts in which model outputs are framed and interpreted in the decision making process.

Given these conclusions, application of the axiom *wrong but useful* as a justification for the use of models in decision-making is highly problematic, as neither the concept of ‘wrong’ nor of ‘useful’ have fixed and definable meanings. At face value this may appear as argument against any role for infectious disease dynamical modelling in decision-making. However, this is not our aim. Rather we agree with Stirling [48] and Leach and Scoones [49] that domains in which ambiguity, uncertainty and ignorance are key features, such as is the case for dynamical modelling of emerging novel epidemics, are better addressed by plural, conditional and nuanced advice, of which infectious disease dynamical modelling may form a (modestly) useful part.
