# Correction: “Wrong, but Useful”: Negotiating Uncertainty in Infectious Disease Modelling

**DOI:** 10.1371/annotation/240b4b75-2b98-4c22-bf3b-f700369f594b

**Published:** 2014-01-03

**Authors:** Robert M. Christley, Maggie Mort, Brian Wynne, Jonathan M. Wastling, A. Louise Heathwaite, Roger Pickup, Zoë Austin, Sophia M. Latham

Links to Figure 1 were incorrectly inserted in the terms "Fig. 1A" and "Fig. 1B" in the extract labeled "E9 (scientific paper)" in the Production of Models section of the Results and Discussion. These terms refer to a figure mentioned in the extract and not Figure 1 of the article. In addition, Figure 1 is placed incorrectly within the article. Rather than immediately below the aforementioned extract, Figure 1 should appear immediately below the first paragraph of the sub-section "Comparison against independent data" in the "Testing Models" section of the Results and Discussion.

